# Quantitative phosphoproteomic analysis reveals unique cAMP signaling pools emanating from AC2 and AC6 in human airway smooth muscle cells

**DOI:** 10.3389/fphys.2023.1149063

**Published:** 2023-02-28

**Authors:** Isabella Cattani-Cavalieri, Yue Li, Jordyn Margolis, Amy Bogard, Moom R. Roosan, Rennolds S. Ostrom

**Affiliations:** ^1^ Department of Biomedical and Pharmaceutical Sciences, Chapman University School of Pharmacy, Irvine, CA, United States; ^2^ Department of Pharmacy Practice, Chapman University School of Pharmacy, Irvine, CA, United States; ^3^ AB Research LLC, Cincinnati, OH, United States

**Keywords:** adenylyl cyclase, cAMP, g-protein coupled receptors, human airway smooth muscle, phosphoproteomics

## Abstract

Human airway smooth muscle (HASM) is the primary target of ßAR agonists used to control airway hypercontractility in asthma and chronic obstructive pulmonary disease (COPD). ßAR agonists induce the production of cAMP by adenylyl cyclases (ACs), activate PKA and cause bronchodilation. Several other G-protein coupled receptors (GPCR) expressed in human airway smooth muscle cells transduce extracellular signals through cAMP but these receptors elicit different cellular responses. Some G-protein coupled receptors couple to distinct adenylyl cyclases isoforms with different localization, partly explaining this compartmentation, but little is known about the downstream networks that result. We used quantitative phosphoproteomics to define the downstream signaling networks emanating from cAMP produced by two adenylyl cyclases isoforms with contrasting localization in uman airway smooth muscle. After a short stimulus of adenylyl cyclases activity using forskolin, phosphopeptides were analyzed by LC-MS/MS and differences between cells overexpressing AC2 (localized in non-raft membranes) or AC6 (localized in lipid raft membranes) were compared to control human airway smooth muscle. The degree of AC2 and AC6 overexpression was titrated to generate roughly equal forskolin-stimulated cAMP production. 14 Differentially phosphorylated proteins (DPPs) resulted from AC2 activity and 34 differentially phosphorylated proteins resulted from AC6 activity. Analysis of these hits with the STRING protein interaction tool showed that AC2 signaling is more associated with modifications in RNA/DNA binding proteins and microtubule/spindle body proteins while AC6 signaling is associated with proteins regulating autophagy, calcium-calmodulin (Ca^2+^/CaM) signaling, Rho GTPases and cytoskeletal regulation. One protein, OFD1, was regulated in opposite directions, with serine 899 phosphorylation increased in the AC6 condition 1.5-fold but decreased to 0.46-fold by AC2. In conclusion, quantitative phosphoproteomics is a powerful tool for deciphering the complex signaling networks resulting from discreet signaling events that occur in cAMP compartments. Our data show key differences in the cAMP pools generated from AC2 and AC6 activity and imply that distinct cellular responses are regulated by these two compartments.

## Introduction

Human airway smooth muscle (HASM) becomes hypercontractile in obstructive lung diseases such as asthma and chronic obstructive pulmonary disease (COPD). Short-acting and long-acting ßAR agonists have been the mainstay of managing airway hypercontractility for decades. These drugs activate ß_2_AR in HASM to stimulate the production of cyclic adenosine monophosphate (cAMP) by adenylyl cyclases (ACs), which activates PKA and leads to bronchodilation (Morgan et al., 2014) but also activates effectors such as EPAC and POPDC (Schmidt et al., 2013; Brand and Schindler, 2017) and can directly regulate cyclic nucleotide gated channels (Rich et al., 2000). Many G-protein coupled receptors (GPCR) expressed in HASM cells utilize cAMP as a primary second messenger, however, agonists for these receptors elicit responses that are distinct from those elicited by ß_2_AR. This phenomenon supports the widely held belief that cAMP signaling is compartmentalized (Baillie, 2009; Houslay, 2010), yet little is known about how cells accomplish this compartmentation (Ostrom et al., 2012). Given that overuse of ß_2_AR agonists is associated with negative disease outcomes (Patel et al., 2013; Reddel et al., 2022), advances in treating asthma and COPD may depend on understanding how cells spatially organize signals.

Most cells express between 100–200 different GPCRs that respond to a wide variety of neurohumoral signals (Insel et al., 2015). From this, it can be predicted that at least a few dozen receptors couple to Gs and the production of cAMP. These facts raise the question, “How do these receptors yield different cellular responses despite using a common second messenger?” It is widely accepted that separate pools, or compartments, of cAMP exist within cells but the specific molecular machinery that establish and maintain these pools are ill-defined. Progress in understanding cAMP compartments was historically limited by the inability to monitor this second messenger in real time and to measure the subcellular location of such signals. FRET- and fluorescent-based sensors that report cAMP in specific locales inside live cells have been developed in recent years (Agarwal et al., 2017; Agarwal et al., 2018) allowing advancement in understanding compartmentalized signaling. However, there is a dearth of information on how signaling proteins downstream of cAMP are stratified between compartments, preventing an understanding of how different cAMP pools relay signals through complex signaling networks to alter cell function and physiology.

Another problem has been the model systems typically used to study molecular signaling. Undifferentiated cells historically used in molecular signaling studies, such as fibroblasts and HEK-293, lack readily defined cellular responses emanating from compartmentalized cAMP signals. Put differently, most cAMP signals lead to relatively homogenous responses in these cells. By contrast, well-differentiated HASM cells have several responses that clearly result from compartmentalized cAMP signaling (Bogard et al., 2012; Horvat et al., 2012; Bogard et al., 2013). These cells provide an excellent model for studying cAMP compartmentation. In this study, we developed a proteomic approach to better understand the signaling by different compartments in HASM.

cAMP compartments are established, in part, by localized AC isoform expression (Ostrom et al., 2000; Ostrom and Insel, 2004). Our data show that in HASM an individual AC isoform generates a cAMP signal that provokes unique cellular responses that signals generated by a different AC isoform cannot induce (Bogard et al., 2013). We also know that specific GPCR couple primarily to co-localized AC isoforms (Johnstone et al., 2018a; Ostrom et al., 2021). In HASM, two cAMP pools compartments have been characterized: ß_2_AR activating AC6 activity and EP_2/4_ receptors coupling to primarily AC2 activity (Bogard et al., 2011; Bogard et al., 2012; Bogard et al., 2013; Agarwal et al., 2017; Agarwal et al., 2019).

Several other molecules play critical roles in establishing cAMP signaling compartments, including phosphodiesterases (PDEs) and A kinase anchoring proteins (AKAPs) (Horvat et al., 2012; Agarwal et al., 2014; Johnstone et al., 2018a). We recently found that one specific PDE isoform regulates cAMP levels in a single compartment (Johnstone et al., 2018b). We still do not know if other PDE isoforms (such as the therapeutically-relevant PDE4 isoforms) have compartment-specific roles (Schmidt et al., 2020). Almost nothing is known about how signaling phosphoproteins downstream of PKA, as well as those altered by cAMP activation of Epac and POPDC (Schmidt et al., 2013; Brand and Schindler, 2017), are regulated by cAMP signals emanating from different signaling pools.

Phosphoproteomics can be leveraged to examine the mechanisms of cAMP action (Beavo et al., 2021) but no studies to date have used such approaches to understand cAMP compartmentation. To address this critical gap in our knowledge, we used Stable Isotope Labeling of Amino acids in Cell culture (SILAC) to quantify phosphorylated proteins in HASM. Phosphopeptides were analyzed by LC-MS/MS and the quantitative difference between light and heavy labeled SILAC pairs represented the change in abundance of that peptide caused by forskolin treatment. Data from AC2- or AC6-overexpressing HASM was analyzed relative to lacZ cells (the control that represents the signaling by all native AC isoforms) to isolate the effect of just the overexpressed AC. The sequences of the phosphopeptides that were differentially regulated in these conditions were analyzed for the associated signaling pathways, known kinase motifs and physiological functions ascribed to these kinases.

## Materials and methods

### Cell culture

(HASM) cells were isolated from non-asthmatic donor lungs. HASM cells were kept at 5% CO_2_ at 37°C, using Ham’s F-12 medium (Thermo Fisher Scientific) supplemented with 10% fetal bovine serum, 1% of antibiotics (penicillin/streptomycin), 25 mM HEPES, 1.7 mM CaCl2, and l-glutamine.

### cAMP assay in live cells

The kinetic cAMP response was measured using the green downward cAMP biosensor (cADDis) (Montana Molecular, Bozeman, MT) in living HASM cells with AC2 or AC6 overexpression. HASM cells were grown on a flask to 60% confluency then plated on a 96-well plate combined with recombinant BacMam virus expressing the cADDis sensor and 1 µM trichostatin-A (Thermo Fisher Scientific). Cells were incubated for 24 h at 37°C and 5% CO_2_ levels. Media was aspirated and replaced with 180 μL per well of 1× Dulbecco’s PBS solution (Gibco) with calcium and magnesium. The 96-well plate was covered with aluminum foil and incubated at room temperature for 30 min. Cells were incubated with vehicle or different concentrations of forskolin; and fluorescence changes were read at 30 s intervals for 20 min on a fluorescence microscope. The fluorescence decay to each forskolin concentration was plotted as ΔF/F_0_ then fit to a one-site decay model. To create a concentration-response curve, the K (decay rate) and plateau response to each forskolin concentration were multiplied together and plotted on a log scale.

### SILAC quantitative phosphoproteomics

For SILAC experiments, cells were labeled by growing in complete medium supplemented with heavy (^13^C_6_) or unlabeled L-Arg and L-Lys (SILAC 2-Plex amino acids, Cambridge Isotope Laboratories). HASM cells were cultured for three passages to allow adaptation and reach the full incorporation of the stable isotope. After the incorporation, the cells were infected with either AdV-lacZ, AdV-AC2 or AdV-AC6 for 24 h. Then, cells were exposed to vehicle or forskolin (1 μM, LC Laboratories) for 10 min at 37°C and cell lysates collected. SILAC pairs were created with the combination of equal amounts of total protein from vehicle-treated light cultures with forskolin-treated heavy cultures. The experiment was repeated in three different HASM cell lines.

Proteins in solution (approximately 1 ug/uL in 2%SDS, 1 mM PMSF, 1 mM EDTA, NaOrthovanadate, NaPyrophosphate, beta glycerophosphate, NaF) were processed as follows. SDS was removed using Pierce detergent removal spin cartridges (#87779, Pierce). Proteins were reduced with 2 mM TCEP at 60°C for 1 h, alkylated with 2 mM MMTS in the dark for 30 min and digested overnight at 37°C by adding 1:5 ratio of Trypsin/LysC mixture (V5071, Promega) to protein in 100 mM TEAB. The resulting peptides were separated into 84 fractions at 250°uL/min using a 0%–90% acetonitrile gradient in 10 mM TEAB on a 150 mm × 2.1 mm ID Waters XBridge 5 um C18 with an Agilent 1,200 capillary HPLC in normal flow mode and Agilent 1,260 micro-fraction collector. The 84 fractions are concatenated into 24 fractions by combining all odd rows of each column 1 through 12 into 12 fractions and all even rows of each column into another 12 fractions. Of the 24 concatenated fractions, 10% was reserved for protein identification, while the remaining 90% was used for phosphopetide enrichment using TiO2 (Larsen et al., 2005).

Peptide fractions were resuspended in 20 uL 2% acetonitrile in 0.1% formic acid and analyzed by reverse phase liquid chromatography coupled to tandem mass spectrometry. Peptides were separated on a 75 um x 150 mm ProntoSIL-120-5-C18 H column (3 µm, 120Å (BISCHOFF), www.bischoff-chrom.com) using 2%–90% acetonitrile gradient at 300 nL/min over 90 min on a an Eksigent nano LC-2D (Sciex.com). Eluting peptides were sprayed through 1 µm emitter tip (New Objective, www.newobjective.com) at 2.0 kV directly into a LTQ Velos Orbitrap (Thermo Scientific) mass spectrometer. Survey scans (full MS) were acquired from 350 to 1800 m/z with data dependent monitoring of up to eight peptide masses (precursor ions), each individually isolated in a 1.2 Da window and fragmented using normalized collision energy of 40. Precursor and the fragment ions were analyzed at resolutions 60,000 and 15,000, respectively. First mass was 110 m/z and lock mass was set to 371.10 Da. Isotopically resolved masses in precursor (MS) and fragmentation (MS/MS) spectra were processed in Proteome Discoverer (PD) software (v1.4, Thermo Scientific). All data were searched using Mascot (2.5.1; www.matrixscience.com) against the Refseq human 2012 database. The following criteria were set for all database searches: human species; trypsin as the enzyme, allowing one missed cleavage; cysteine carbamidomethylation as fixed modification; with heavy lysine and arginine, methionine oxidation, asparagine and glutamine deamidation as variable modifications. Peptide identifications from Mascot searches were filtered at 1% False Discovery Rate (FDR) confidence threshold, based on a concatenated decoy database search, using PD. The PhosphoRS node in PD calculated the probability of phosphorylation for each Ser/Thr/Tyr site. Heavy/light ratios for peptides/proteins were calculated using the quantitation node of PD.

Light/heavy ratios were combined into matrices for both protein and peptide level analyses. The light/heavy ratios were normalized using a median_normalization () function from proDA package. Then, data were log2 transformed for further analysis. Based on treatment conditions, we evaluated differentially expressed proteins and peptide separately. Proteins with fold change of 1.5 or 1/1.5 of the light/heavy ratio and *p*-value <0.05 were considered differentially expressed while peptides with fold change of 1.5 or 1/1.5 of the light/heavy ratio and false discovery rate <0.05 with were considered differentially expressed. GeneBank protein ID numbers were converted to HGNC gene symbols using the UniProt.org (Nucleic Acids Res. 49:D1 (2021), accessed on 06/29/22). Gene Enrichment analysis was completed using the Molecular Signature Database gene sets. Packages used are limma_3.42.2, proDA_1.0.0, msigdbr_ 7.5.1, fgsea_1.12.0, and dplyr_1.0.9 on R version 3.6.2 (2019-12-12).

## Results

**FIGURE 1:**
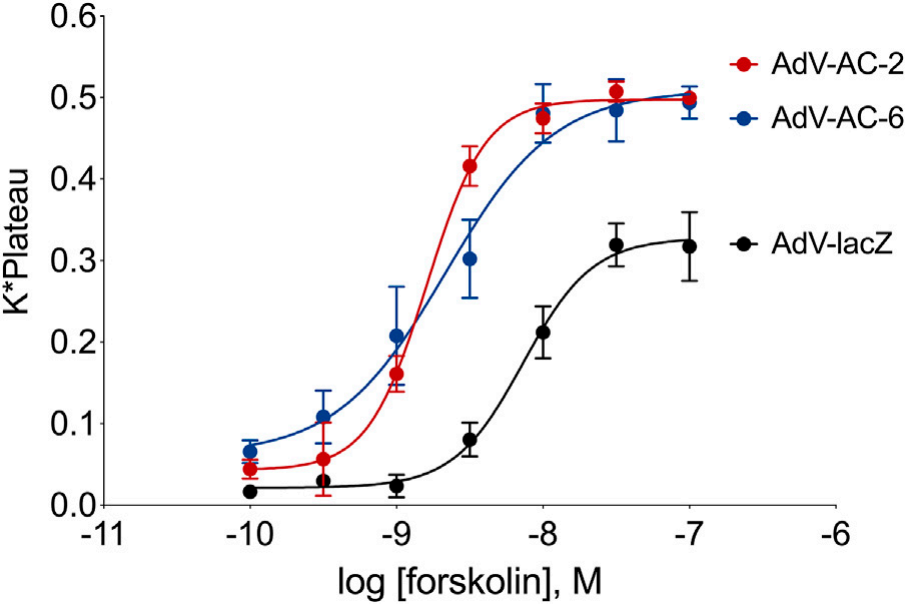
cAMP responses measured using the cADDis biosensor in HASM with AC2 or AC6 overexpression. Cells were incubated for 24 h with the indicated recombinant adenovirus and the cADDis baclovirus for 24 h. Fluorescent decay curves were measured for 20 min following addition of either vehicle (not shown) or various concentrations of forskolin. The response to each forskolin concentration was fit to a one-site decay model and the K (decay rate) and plateau for each concentration were multiplied together and plotted on a log scale. The resultant concentration-response curves were fit with non-linear regression. The data represent the mean ± SEM of three different HASM cell lines.

To make a fair quantitative comparison of phosphopeptides regulated by AC2 and AC6, we first optimized the adenovirus titers to obtain a level of overexpression of each AC isoform that leads to equivalent levels of forskolin-stimulated cAMP. After infecting cells with different titers of each AdV-AC2 and AdV-AC6 we measured cAMP responses to increasing concentrations of forskolin using cADDis, a cAMP biosensor. We chose titers of each adenovirus that gave roughly equal leftward shifts in EC_50_ and equal increases in E_max_ as compared to AdV-lacZ infected cells (Figure 1). Forskolin-stimulated responses in AdV-lacZ infected HASM displayed a logEC_50_ of −8.14 ± 0.04 and an E_max_ of 0.328 ± 0.010 irrespective of the titer used. After infection of HASM with AdV-AC2, forskolin was more potent and more efficacious, with a logEC_50_ of −8.79 ± 0.02 and an E_max_ of 0.498 ± 0.007. After infection of HASM with AdV-AC6, forskolin potency and efficacy was similarly enhanced, with a logEC_50_ of −8.67 ± 0.12 and an E_max_ of 0.509 ± 0.030. AC2 and AC6 immunoreactivity were similarly increased, and each detected protein was appropriately localized as compared to native expression in lipid raft and non-raft cellular fractions (not shown) (Bogard et al., 2012).

We then grew HASM cells in the presence of either heavy or light amino acids for three passages, infected them with either AdV-lacZ, AdV-AC2 or AdV-AC6 for 24 h. Cells were then exposed to either vehicle or forskolin (1 µM) for 10 min at 37°C and whole cell lysates collected. SILAC pairs were created by combining equal amounts of total protein from vehicle-treated light cultures with forskolin-treated heavy cultures (or visa-versa to avoid heavy-light bias). Samples were trypsin digested, enriched for phosphopeptides on a TiO_2_ column then analyzed by LC-MS/MS. Quantitative differences between light and heavy labeled SILAC pairs represented the change in abundance of that peptide caused by forskolin treatment. 7,000–10,000 phosphopeptides (2,100-2,500 unique proteins) were detected in each sample. This experiment was repeated in three different HASM cell lines.

HASM natively express multiple AC isoforms, including AC2 and AC6. To determine the phosphoproteins regulated by a single AC isoform one must filter out the signaling initiated from the complement of native ACs. To achieve this, we subtracted the effects of forskolin in lacZ (control) cells from each of the AC overexpressing conditions, leaving only phosphorylation events that were enhanced by AC2 overexpression or AC6 overexpression, respectively. HSP20 is a known target of phosphorylation by PKA (Tyson et al., 2008; Edwards et al., 2012). We observed that HSP20 phosphorylation on ser16 was increased by forskolin treatment 8.99 ± 2.86 fold in lacZ cells, 9.64 ± 4.99 fold in AC2 overexpressing cells and 12.36 ± 5.71 fold in AC6 overexpressing cells. HSP20 didn’t emerge in our list of significantly phosphorylated peptides. This may be due to the variability of the response we observed and the relatively strict *p*-value cutoff we used. It may also be due to a stoichiometric limitation in PKA phosphorylation of HSP20 such that AC overexpression is not able to increase signaling in this pathway. Non-etheless, the observed regulation of HSP20 serves as internal validation of the methodology used.

**FIGURE 2:**
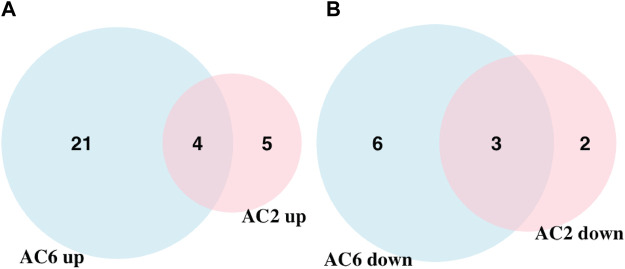
Venn diagram showing the overlap of up and downregulated differentially phosphorylated proteins in AC2 and AC6 samples. **(A)** 25 and nine upregulated proteins were detected compared to control samples whereas **(B)** nine and five downregulated proteins were detected in AC6 and AC2 samples, respectively. Only four and three proteins were common between the two groups in up and downregulated proteins. The rest of the detected proteins were unique to each treatment condition. Overall, twice as many differentially phosphorylated proteins were identified in AC6 samples compared to AC2 samples.

We applied statistical analyses to determine the significantly altered phosphoproteins across our three experiments without concern for the kinases or signaling pathways that may have led to a particular change in phosphorylation. The changes in phosphopeptide levels may or may not result directly from PKA signaling. Proteins with fold change greater than 1.5 or less than 1/1.5 of the light/heavy ratio with *p*-values <0.05 were considered differentially phosphorylated. We identified a total of 14 DPPs associated with AC2 signaling (Table 1) and 34 DPPs associated with AC6 signaling (Table 2). Remarkably, just four up-phosphorylated proteins and three down-phosphorylated proteins were the common between the AC2 and AC6 pools (Figure 2; Tables 3, 4).

**TABLE 1:** List of differentially phosphorylated proteins in AC2 versus control. Proteins with fold change greater than 1.5 or less than 1/1.5 of the light/heavy ratio with *p*-values <0.05 were considered differentially phosphorylated. Fourteen such proteins were identified in the AC2 samples.

Proteins	Gene symbol	Log2 fold change	*p*-value
misshapen-like kinase 1 isoform 4	MINK1	2.42923292	0.01323219
serine/threonine-protein kinase Nek4 isoform 2	NEK4	1.82308983	0.00815235
kinesin light chain 4 isoform a	KLC4	1.47509245	0.01108237
oxysterol-binding protein-related protein 5 isoform b	OSBPL5	1.20267437	0.00095095
cell surface glycoprotein MUC18 precursor CD146	MCAM	1.14932511	0.03059349
putative ATP-dependent RNA helicase DHX57	DHX57	0.83329342	0.02529122
tissue factor pathway inhibitor 2 precursor	TFPI2	0.72935507	0.03062731
protein unc-93 homolog B1	UNC93B1	0.63058874	0.03772098
GRB2-associated-binding protein 2 isoform b	GAB2	0.60937488	0.04827974
eukaryotic elongation factor 2 kinase	EEF2K	−0.9400986	0.0243547
growth factor receptor-bound protein 10 isoform c	GRB10	−0.9826081	0.01994397
SUN domain-containing protein 1 isoform e	SUN1	−0.9897727	0.01973597
oral-facial-digital syndrome 1 protein	OFD1	−1.1284668	0.01633036
layilin isoform 3	LAYN	−1.2519656	0.01405283

**TABLE 2:** List of differentially phosphorylated proteins in AC6 versus control. Proteins with fold change greater than 1.5 or less than 1/1.5 of the light/heavy ratio with *p*-values <0.05 were considered phosphorylated expressed. Thirty-four such proteins were identified in the AC6 samples.

Protein	Gene symbol	Log2 fold change	*p*-value
death-associated protein kinase 2	DAPK2	3.7543113	0.0028564
serine/threonine-protein kinase Nek4 isoform 2	NEK4	2.83334994	0.00429885
zinc finger protein GLI2	GLI2	2.03687387	0.00694102
TBC1 domain family member 9B isoform b	TBC1D9B	1.60021883	0.03226196
RING finger protein 169	RNF169	1.55459132	0.01027069
kinesin light chain 4 isoform a	KLC4	1.39370357	0.01203196
rho GTPase-activating protein 11A isoform 2	ARHGAP11A	1.24847298	0.01410975
leucine-rich repeat and calponin homology domain-containing protein 3 precursor	LRCH3	1.23717817	0.01429651
torsin family protein C9orf167	TOR4A	1.07906994	0.00146051
calmodulin-regulated spectrin-associated protein 1	CAMSAP1	0.93032508	0.02953968
ras-associated and pleckstrin homology domains-containing protein 1 isoform 1	RAPH1	0.92963962	0.02160351
serine/threonine-protein kinase SIK3	SIK3	0.86055831	0.02414587
rho GTPase-activating protein 29	ARHGAP29	0.80633695	0.02651636
putative oxidoreductase GLYR1	GLYR1	0.78646216	0.02748503
tissue factor pathway inhibitor 2 precursor	TFPI2	0.76998971	0.02833385
KAT8 regulatory NSL complex subunit 1 isoform 2	KANSL1	0.72989357	0.00242888
starch-binding domain-containing protein 1	STBD1	0.72394533	0.03095616
calcium/calmodulin-dependent protein kinase kinase 2 isoform 6	CAMKK2	0.70648771	0.03205888
NHS-like protein 1 isoform 2	NHSL1	0.70426944	0.03220372
BCL2/adenovirus E1B 19 kDa protein-interacting protein 3	BNIP3	0.66455668	0.03499499
zinc finger CCCH domain-containing protein 11A	ZC3H11A	0.60668636	0.02898325
protein FAM101B	RFLNB	0.6009773	0.04040135
oral-facial-digital syndrome 1 protein	OFD1	0.60012471	0.04048322
protein unc-93 homolog B1	UNC93B1	0.59718445	0.04076771
rho GTPase-activating protein 31	ARHGAP31	0.59646839	0.04326041
growth factor receptor-bound protein 10 isoform c	GRB10	−0.6143478	0.03915281
receptor-interacting serine/threonine-protein kinase 3	RIPK3	−0.6911084	0.03308611
rap guanine nucleotide exchange factor 6 isoform 5	RAPGEF6	−0.7522246	0.0292998
PERQ amino acid-rich with GYF domain-containing protein 2 isoform b	GIGYF2	−0.7885298	1.41E-05
eukaryotic elongation factor 2 kinase	EEF2K	−0.7919509	0.04910675
epidermal growth factor receptor kinase substrate 8	EPS8	−0.8618852	0.02409237
SUN domain-containing protein 1 isoform e	SUN1	−0.8764269	0.02351879
rho-associated protein kinase 1	ROCK1	−0.8854265	0.0012546
Pericentrin	PCNT	−0.8860854	0.02315039

**FIGURE 3:**
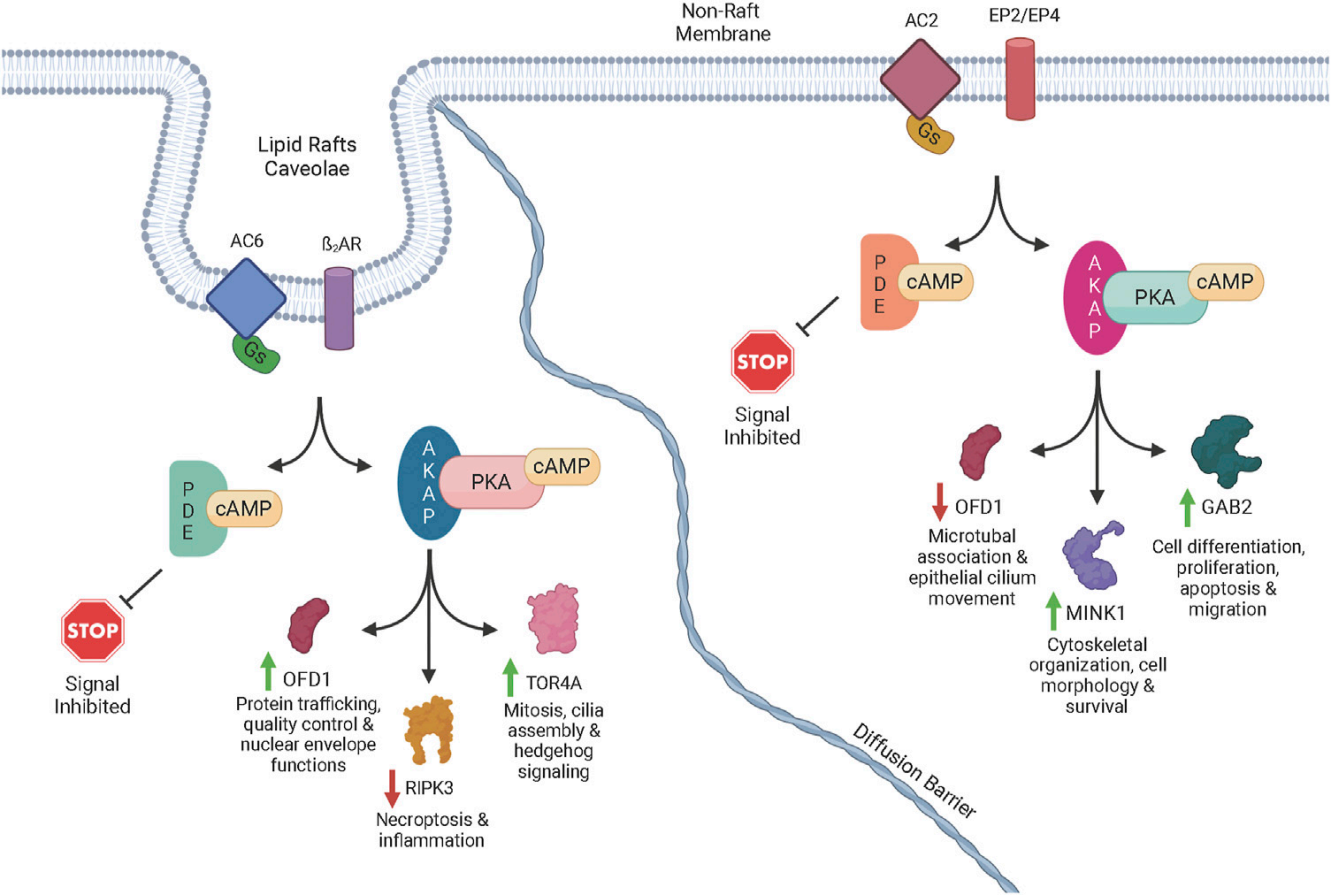
Schematic diagram of cAMP signaling *via* transmembrane ACs. Once activated by forskolin or a GPCR agonist acting through Gs, an AC isoform catalyzes the reaction of ATP to cAMP. The cAMP signal is terminated though the activity of one or more PDEs that degrade cAMP. The cAMP signal is advanced though the activation of PKA, Epac, and POPDC. When PKA is activated by cAMP, AKAP-directed targeting directs phosphorylation of specific proteins to generate signals through distinct pathways. POPDC and EPAC also initiate signaling and, in addition to the PKA-initiated signals, generate complex signaling networks *via* crosstalk between pathways. The complex signaling events can lead to increased or decreased phosphorylation of various proteins. For example, lipid raft AC6 led to increased phosphorylation of OFD1 (green arrow) potentially producing physiological effects such as mitosis, cilia assembly and hedgehog signaling. However, non-raft localized AC2 led to decreased phosphorylation of OFD1 (red arrow) potentially causing different effects such as microtubule association and epithelial cilium movement. A few of the other phosphoproteins that were increased or decreased by a specific AC signaling pool in our studies are shown along with some of the physiological effects associated with those proteins.

**TABLE 3:** List of common and differentially increased phosphorylated proteins by AC2 or AC6. Proteins with fold change of 1.5 or greater of the light/heavy ratio and *p*-value <0.05 were considered differentially phosphorylated.

AC2 unique upregulated proteins	AC6 unique upregulated proteins	Common upregulated proteins
MINK1	DAPK2	NEK4
OSBPL5	GLI2	KLC4
MCAM	TBC1D9B	TFPI2
DHX57	RNF169	UNC93B1
GAB2	ARHGAP11A	
	LRCH3	
	TOR4A	
	CAMSAP1	
	RAPH1	
	SIK3	
	ARHGAP29	
	GLYR1	
	KANSL1	
	STBD1	
	CAMKK2	
	NHSL1	
	BNIP3	
	ZC3H11A	
	RFLNB	
	OFD1	
	ARHGAP31	

**TABLE 4:** List of common and differentially decreased phosphorylated proteins by AC2 or AC6. Proteins with fold change less than 1/1.5 of the light/heavy ratio and *p*-value <0.05 were considered differentially phosphorylated.

AC2 unique downregulated proteins	AC6 unique downregulated proteins	Common downregulated proteins
LAYN	RIPK3	GRB10
**OFD1**	RAPGEF6	EEF2K
	GIGYF2	SUN1
	EPS8	
	ROCK1	
	PCNT	

Using the differentially phosphorylated proteins identified, we performed a Gene Set Enrichment Analysis (GSEA). GSEA identified statistically significant pathways with a *p*-value <0.05 reflected with enrichment scores. Enrichment score indicates the degree to which a pathway is overrepresented at the top or bottom of the ranked list of phosphorylated proteins. This analysis makes the assumption that a change in phosphorylation of a protein represents a signal within the network(s) in which that protein is associated. 11 and 78 pathways were significant using AC2 and AC6 (Supplemental Tables S1, S2) differentially regulated proteins, respectively. We also used STRING to categorize the differentially phosphorylated proteins regulated by each cAMP signaling pool. The STRING tool contains both known and predicted protein associations based on protein-protein interactions and functional relationships (Szklarczyk et al., 2021). While several pathways are similar, AC2 signaling is more strongly associated with alterations in RNA/DNA binding proteins and microtubules/spindle body proteins (Table 5), while AC6 signaling is strongly associated with proteins regulating autophagy, Ca^2+^/CaM signaling, Rho GTPases and cytoskeletal regulation (Table 6).

**TABLE 5:** Proteins and peptides with increased or decreased phosphorylation from AC2 and associated kinases and pathways. The amino acid that was phosphorylated in each sequence is underlined.

Gene symbol	Phosphorylated sequence	Potential kinase	Pathway (from STRING)
MINK1	KGSVVNVNPTNTR	PKA, PKC*α*, *β*, *γ*	KOG0587—Traf2- and Nck-interacting kinase and related germinal center kinase family protein kinases
			COG1100—GTPase SAR1 family domain
NEK4	ASLSVAGPGKPQEEDQPLPAR	PKA, Aurora B, PKC*δ*	KOG0589—Serine/threonine protein kinase
KLC4	RSSELLVR	PKA, Aurora A/B, PKCμ, 14-13-3 mode 1	COG0457—Tetratricopeptide (TPR) repeat
OSBPL5	RFSLCPPSSTPQK	PKA AKT, AMP, Aurora A, Aurora B, 14-3-3 Mode 1	KOG2210—Oxysterol-binding protein
MCAM	KSELVVEVK	None found	NOG259118—vascular wound healing
DHX57	DLQEQDADAGSERGLSGEEE	CK1, CK2	COG1643—HrpA-like RNA helicase
TFPI2	DEGLCSANVTR	ATK, ERK1, GSK3, NEK6	KOG4295—Serine proteinase inhibitor (KU family)
UNC93B1	YLEEDNSDESDAEGEHGDGA	None found	KOG3097—Predicted membrane protein
GAB2	RNTLPAMDNSR	PKA, Aurora A/B, AMPK, Akt, 14-3-3 Mode 1	NOG05681—transmembrane receptor protein tyrosine kinase adaptor activity
EEF2K	KYESDEDSLGSSGR	None found	NOG07806—Eukaryotic elongation factor 2 kinase
			KOG0598—Ribosomal protein S6 kinase and related proteins
GRB10	SIQPQVSPR	CDK5, CDK2, CDK1	KOG3751—Growth factor receptor-bound proteins (GRB7, GRB10, GRB14)
			KOG1862—GYF domain containing proteins
SUN1	RPPVLDESWIR	CDK5, CDK2, CDK1, Nek6, Nek7, PLK1	KOG2687—Spindle pole body protein, contains UNC-84 domain
			NOG269324—microtubule cytoskeleton organization involved in homologous chromosome segregation
OFD1	RQSNLQEVLER	PKA, Aurora A/B, CDC2, CLK2, CDK1, CDK2, CDK5, Erk1, GSK3	NOG258115—epithelial cilium movement involved in determination of left/right asymmetry
			KOG1654—Microtubule-associated anchor protein involved in autophagy and membrane trafficking
LAYN	KQSEADLAETRPDLK	PKA, AMPK, Aurora B	KOG4297—C-type lectin

**TABLE 6:** Proteins and peptides with increased or decreased phosphorylation from AC6 and associated kinases and pathways. The amino acid that was phosphorylated in each sequence is underlined.

Gene symbol	Phosphorylated sequence	Potential kinase	Pathway (from STRING)
DAPK2	KASVVDPSTESSPAPQEGSEQ	PKA, AMPK, Aurora A/B, Akt	KOG0032- Ca2+/calmodulin-dependent protein kinase
NEK4	ASLSVAGPGKPQEEDQPLPAR	PKA, Aurora A/B, Akt, CaMKII, CDK2, CDK5, CLK2, ERK1, PKC*δ*, PKC*ε*	KOG0589—Serine/threonine protein kinase
GLI2	RDSSTSTVSSAYTVSR	PKA, AMP, Aurora A/B, Akt, AMPK, CLK2, CK1, GSK3, PKCμ, PKCε	KOG3599—Ca2+-modulated non-selective cation channel polycystin
			KOG3577—Smoothened and related G-protein-coupled receptors
			KOG0595—Serine/threonine-protein kinase involved in autophagy
			COG5059—microtubule motor activity
TBC1D9B	KASVVDPSTESSPAPQEGSEQ	None Found	COG5210—TBC1 domain family member
RNF169	KGSVDQYLLR	None Found	COG2802—Uncharacterized protein, similar to the N-terminal domain of Lon protease
KLC4	RSSELLVR	PKA, AMPK, Akt, Aurora A/B, CaMKII, CLK2, NEK3, NEK4, PKC, PKCμ	COG0457—Tetratricopeptide repeat
ARHGAP11A	RQSVGDFVSGALNK	NEK2, NEK6, NEK7, NEK 9, NEK10, PLK1	KOG2710—Rho GTPase-activating protein
LRCH3	RESQYQENR	PKA, Aurora A/B, ATM, Akt	COG4886—Leucine-rich repeat (LRR) protein
TOR4A	SRLVLYPETSR	PKA, Akt, Aurora A/B, CLK2, NEK1, NEK4, GSK3, PKC	KOG2170—ATPase of the AAA + superfamily
CAMSAP1	RPSEGPQPLVR	None Found	KOG3654—Uncharacterized CH domain protein
RAPH1	RPSVDSLVSK	PKA, Aurora A/B, CaMK, GSK3, AMPK, DNA-PK, CDK1, CDK2, CDK5, CK2	KOG3751—Growth factor receptor-bound proteins (GRB7, GRB10, GRB14)
			COG0515—Serine/threonine protein kinase
SIK3	HSLTGHSDIR	PKA, Aurora A/B, NEK3	KOG0586—Serine/threonine protein kinase
			KOG0583—Serine/threonine protein kinase
ARHGAP29	RSSDSYPLAPVR	CDK1, CDK2, CDK5	KOG1453—Chimaerin and related Rho GTPase activating proteins
			COG5022—Myosin heavy chain
GLYR1	KLSLSEGK	Akt, Aurora A/B, CaMK, PKCε	COG2084—3-hydroxyisobutyrate dehydrogenase or related beta-hydroxyacid dehydrogenase
			KOG1904—Transcription coactivator
TFPI2	DEGLCSANVTR	ATK, ERK1, GSK3, NEK6	KOG4295—Serine proteinase inhibitor (KU family)
KANSL1	LSPGTDSSSNLGGVK	CDK1 motif 1 and 2, Cdc2, Cdk5, Erk1, GSK3	NOG10191—histone H4-K16 acetylation
STBD1	HSSWGDVGVGGSLK	Akt, Aurora A/B, CLK2	NOG41395—glycogen binding
			NOG253677—FAM47 family
CAMKK2	SLSAPGNLLTK	None found	KOG0585—Ca2+/calmodulin-dependent protein kinase kinase beta and related serine/threonine protein kinases
NHSL1	NSGAEAAQLSER	Erk1, GSK3	NOG254239—motor neuron migration
BNIP3	NSSQSEEDDIER	DNA-PK, CK2	NOG252945—mitochondrial protein catabolic process
			COG0183—Acetyl-CoA acetyltransferase
ZC3H11A	RLSSASTGKPPLSVEDDFEK	None found	KOG4791—Uncharacterized conserved protein
RFLNB	LSLQDVPELVDAK	None found	NOG14684—regulation of chondrocyte development
OFD1	RQSNLQEVLER	PKA, Aurora A/B, CDK1, CDK2, CLK2, CDK5, Erk1, GSK3	NOG258115—epithelial cilium movement involved in determination of left/right asymmetry
			KOG1654—Microtubule-associated anchor protein involved in autophagy and membrane trafficking
UNC93B1	YLEEDNSDESDAEGEHGDGA	None found	KOG3097—Predicted membrane protein
ARHGAP31	RNSAPVSVSAVR	None found	KOG1449—Predicted Rho GTPase-activating protein CdGAPr
			COG5422—Rho guanyl-nucleotide exchange factor activity
			KOG1029—Endocytic adaptor protein intersectin
GRB10	MNILGSQSPLHPSTLSTVIHR	AMP, CDC2, CDK1, CDK2, CDK5, ERK1, P38 MAPK	KOG3751—Growth factor receptor-bound proteins (GRB7, GRB10, GRB14)
			KOG1862—GYF domain containing proteins
RIPK3	NQMPSPTSTGTPSPGPR	PKA, Akt, Aurora A/B, CaMK, CLK2, PKC*α*, *β*, *γ*, *ε*	COG0515—Serine/threonine protein kinase
			NOG60689—left-handed Z-DNA binding
RAPGEF6	SSEMSPVPMR	None found	KOG3629—Guanine-nucleotide releasing factor
GIGYF2	WRPHSPDGPR	None found	KOG1862—GYF domain containing proteins
EEF2K	LEGVDGGQSPR	None found	NOG07806—Eukaryotic elongation factor 2 kinase
			KOG0598—Ribosomal protein S6 kinase and related proteins
EPS8	DSVSSVSDISQYR	Aurora A/B	KOG3557—Epidermal growth factor receptor kinase substrate
			NOG05299—SCF-dependent proteasomal ubiquitin-dependent protein catabolic process
			KOG2546—Abl interactor ABI-1, contains SH3 domain
SUN1	RPPVLDESWIR	None found	NOG269324—microtubule cytoskeleton organization involved in homologous chromosome segregation
			KOG2687—Spindle pole body protein, contains UNC-84 domain
ROCK1	LLDLSDSTSVASFPSADET	NEK6, NEK7	COG0515—Serine/threonine protein kinase
			KOG4807—F-actin binding protein, regulates actin cytoskeletal organization
			KOG3530—FERM domain protein EHM2
PCNT	LAAAASPHSGGR	CDK1, PKC*α*, *β*, *δ*, *γ*	NOG07423—protein-containing complex scaffold activity
			NOG258331—interkinetic nuclear migration
			NOG234074—RS domain binding

One protein, OFD1, was regulated in opposite directions by AC2 and AC6 pools. In both conditions, forskolin led to increased phosphorylation of serine 899. However, AC6 increased forskolin-stimulated ser899 phosphorylation 1.5-fold while AC2 decreased it 0.46-fold (Tables 1, 2). The phosphorylated peptide sequence, RQSNLQEVLER, is 100% conserved across 100 species (as per BLAST analysis). Scansite4 predicts that the peptide sequence containing ser899 could be phosphorylated by PKA or Aurora kinase B. Published data sets indicate that OFD1 ser899 phosphorylation is altered by inhibitors of Aurora kinase or Polo-like kinase 1 (PLK1, an early trigger for G2/M transition and can be regulated by PKA (Zhu et al., 2010). These opposite effects of AC2 and AC6 overexpression make clear that the signaling pathways regulating OFD1 phosphorylation are complex and that our methodology doesn’t only reveal effects of direct PKA activity.

## Discussion

Specific isoforms of AC have distinct physiological roles *via* both unique expression patterns throughout the body and by existing in specific subcellular compartments and coupling to specific GPCR (Ostrom et al., 2021). AC2 is expressed in airway smooth muscle and shown to regulate airway contractile tone in response to prostaglandins (Bogard et al., 2011). AC6 is highly expressed in both human and murine airway smooth muscle where it links ß_2_AR to bronchodilation (Bogard et al., 2011; Birrell et al., 2015), however it is also highly expressed in heart, brain, kidney, pancreas, and bones (Ostrom et al., 2021). How AC isoforms regulate other physiological functions of airway smooth muscle is poorly understood. In the present study, we investigated the downstream signaling of cAMP emanating from AC2 and AC6 using quantitative phosphoproteomics. AC2 overexpression led to regulation of proteins associated with cytoskeletal organization, cell morphology, differentiation/proliferation, apoptosis, and migration. By contrast, AC6 overexpression regulated proteins associated with cell cycle regulation, autophagy, cell proliferation and cytoskeleton regulation.

Our data reveal the specific regulation of several proteins by AC2 or AC6. AC2 uniquely increased or decreased phosphorylation of several specific proteins. MINK1 is a member of the protein kinase superfamily and is known to regulate cytoskeletal organization, cell morphology and survival. Oxidative stress is important for the activation of MINK downstream of Ras signaling (Nicke et al., 2005). GAB2 participates in several physiological processes, such as cell differentiation and proliferation, apoptosis, and migration. GAB2 is a scaffold protein that binds to multiple receptors, playing a role in several signal transduction pathways, such as the PI3 kinase, SHP2 and JAK/STAT pathways (Liu and Rohrschneider, 2002; Nyga et al., 2005; Vaughan et al., 2011).

AC6 regulated phosphorylation of a different set of proteins. DAPK2 (also known as DRP-1) is a (Ca^2+^/CaM)-dependent protein kinase that participates in apoptotic signals, autophagy, granulocyte differentiation and motility regulation (Horvath et al., 2021). LRCH proteins are cytoskeletal regulators and linked to the regulation of cell proliferation (Riviere et al., 2020). TOR4A, TorsinA, is a member of the AAA + ATPase family that is located within the lumen of the endoplasmic reticulum and nuclear envelope. AAA + proteins are associated to several biological functions, such as vesicle fusion, cytoskeleton dynamics, intracellular trafficking, and protein folding (Serrano et al., 2019). TorsinA is linked to regulation of protein trafficking quality control and nuclear envelope function (Gonzalez-Alegre and Paulson, 2004; Nery et al., 2011). RAPH1 encodes the Lamellipodin protein, which plays an important role in the regulation of actin dynamics for cytoskeleton regulation (Batistela et al., 2013). SIK3, Serine/threonine-protein kinase is linked to several processes such as cell cycle regulation, gluconeogenesis, and lipogenesis regulation. Salt-inducible kinases (SIKs) are serine/threonine-protein kinases and PKA can phosphorylate them, leading to 14-3-3 protein binding and an inhibition of their catalytic activity (Sonntag et al., 2018). RIPK3, RIPK3 is an essential regulator of necroptosis and modulates inflammation independent of necroptosis (Moriwaki and Chan, 2017). When activated, RIPK3 phosphorylates the mixed lineage kinase domain-like pseudokinase, which is known as terminal pathway effector (Meng et al., 2021). RIPK3 has been observed in complex with mixed lineage kinase domain-like in the cytoplasm before the initiation of necroptosis (Garnish et al., 2021).

Our data shows the opposite regulation of OFD1 by AC2 and AC6. OFD1 localizes to centrioles and pericentriolar satellites and is involved in the formation of primary cilia (Pezzella et al., 2022). Moreover, previous studies have demonstrated that OFD1 plays a role as a novel receptor in autophagy (Tang et al., 2013; Morleo et al., 2021; Morleo and Franco, 2021). Thus, AC2 and AC6 may lead to opposite effects on these cellular functions. These differences may be the reason by which EP receptor and ßAR agonist induces distinct cellular effects despite both signaling through increases in cAMP.

When we examined the phosphorylated peptide sequences and queried for kinases capable of phosphorylating each site, some interesting trends emerged. AC2 overexpression led to more kinases with decreased activity while AC6 overexpression led to more kinases with increased activity (Tables 5, 6). Several the kinases upregulated by AC6, such as aurora A, aurora B and CLK2, are associated with cell cycle regulation (He et al., 2008; Gully et al., 2012; Park et al., 2016). Thus, it is tempting to hypothesize that AC2 and AC6 cAMP signaling pools promote opposite effects on cell cycle regulation in airway smooth muscle. Regardless, it is clear that the AC2 signaling pool is associated with different signaling networks.

GSE A is a valuable tool for identifying enrichment in pathways from gene expression because it can translate changes in many gene products into alterations in signaling pathways (Subramanian et al., 2005). We adapted use of this tool to identify pathways based on the unique phosphoproteins we identified that are regulated by AC2 or AC6 (Supplementary Table S1). While GSEA is typically queried with data on alterations in gene expression, the tool is equally useful for identifying pathways altered in phosphoproteomic data. We used the STRING tool to associate our observed alterations in phosphoproteins with known and predicted protein interactions and to link them to a specific biological function (Szklarczyk et al., 2019). The combination of both tools provides information about the networks of differently regulated proteins associated with signaling *via* AC2 or AC6 and the potential effects of these two cAMP signaling pools on cell physiology.

There are some limitations to our study that should be noted. First, our study was limited to three HASM cell lines from patients of unknown age, gender or race. A larger n size may yield significant differences in more phosphoproteins and pathways. Second, the changes in phosphoproteins we observe are the net result of signaling through many pathways in addition to complex cross-talk between pathways and many negative and positive feedback mechanisms. It is not possible to connect PKA (or Epac or POPDC) activity directly to the phosphorylation events we observe since we did not manipulate expression or activity of these effectors. In addition, overexpression of an AC may not lead to more protein phosphorylation due to stoichiometric limits in the pathway. These cases would be missed using our experimental approach so one must be careful in concluding that pathways we did not observe in this study aren’t associated with a specific signaling compartment. Knockout of AC isoform expression would be a complementary approach to the one utilized here, albeit with its own potential shortcomings, and would yield additional information on how each cAMP pool regulates protein phosphorylation. One shouldn’t make assumptions about phosphorylation stoichiometry having proportional effects on cellular function since some phosphopeptides may be more tightly coupled to changes in cell function than others (Beavo et al., 2021). Further studies are needed to examine the linkage between the phosphoproteins we identify and any physiological effects.

In summary, our study demonstrates that quantitative phosphoproteomics is a powerful tool for understanding cAMP compartmentation. It can help decipher the complex signaling networks that are involved in transducing signaling events from GPCR-Gs-AC complexes at the cell membrane and identify how these effects might alter cell physiology. Our study is a first step in understanding how distinct cAMP signaling compartments lead to unique effects on cell function.

## Data Availability

The datasets presented in this study can be found in online repositories. The names of the repository/repositories and accession number(s) can be found in the article/Supplementary Material. The dataset is also available in Proteome Xchange under accession number PXD039843.

## Abbreviations

AC, Adenylyl cyclase; AKAP, A Kinase Anchoring Protein; Ca^2+^/CaM, Calcium-calmodulin; cAMP, Cyclic Adenine Monophosphate; DPP, Differentially Phosphorylated Proteins; GPCR, G-Protein Coupled Receptor; GSEA, Gene Set Enrichment Analysis; HASM, Human Airway Smooth Muscle; PDE, Phosphodiesterase; PKA, Protein Kinase A; PLK1, Polo-like kinase 1; SIK, Salt-inducible kinases: A family of Kinases that is PKA mediated; SILAC, Stable Isotope Labeling of Amino acids in Cell culture.
