# Supplementary material for: Quantitative phosphoproteomic analysis reveals unique cAMP signaling pools emanating from AC2 and AC6 in human airway smooth muscle cells
Source: Front Physiol. 2023 Feb 28;14:1149063. doi: 10.3389/fphys.2023.1149063 (PMC10011497; doi:10.3389/fphys.2023.1149063)
Supplement: Supplementary file 1 [file Table1.DOCX]

**Supplemental Table 1: Pathway analysis with AC2 differentially phosphorylated proteins.** Gene Set Enrichment analysis identified statistically significant pathways with a p-value <0.05. Enrichment score reflects the degree to which a pathway is overrepresented at the top or bottom of the ranked list of protein. Gene Enrichment analysis was completed using the Molecular Signature Database all protein sets. 11 pathways were significantly enriched for AC2 differentially regulated proteins.

|  | **Enrichment score** | **Normalized Enrichment score** | **p value** |
| --- | --- | --- | --- |
| MIR153_5P | -1 | -1.7626758 | 0.0208605 |
| GOBP_REGULATION_OF_CELLULAR_RESPONSE_TO_STRESS | 1 | 1.68582911 | 0.0233088 |
| GOBP_REGULATION_OF_RESPONSE_TO_STRESS | 1 | 1.68582911 | 0.0233088 |
| GOMF_PROTEIN_SERINE_KINASE_ACTIVITY | 1 | 1.68582911 | 0.0233088 |
| GOMF_PROTEIN_SERINE_THREONINE_TYROSINE_KINASE_ACTIVITY | 1 | 1.68582911 | 0.0233088 |
| GSE22140_HEALTHY_VS_ARTHRITIC_MOUSE_CD4_TCELL_DN | 1 | 1.68582911 | 0.0233088 |
| OSMAN_BLOOD_CHAD63_KH_AGE_18_50YO_HIGH_DOSE_SUBJECTS_24HR_DN | -0.8181818 | -1.6560427 | 0.02511737 |
| GOMF_KINASE_ACTIVITY | 0.81894794 | 1.54011609 | 0.04190412 |
| GOMF_PROTEIN_KINASE_ACTIVITY | 0.81894794 | 1.54011609 | 0.04190412 |
| GOMF_PROTEIN_SERINE_THREONINE_KINASE_ACTIVITY | 0.81894794 | 1.54011609 | 0.04190412 |
| GOMF_TRANSFERASE_ACTIVITY_TRANSFERRING_PHOSPHORUS_CONTAINING_GROUPS | 0.81894794 | 1.54011609 | 0.04190412 |

**Supplemental Table 2: Pathway analysis with AC6 differentially phosphorylated proteins.** Gene Set Enrichment analysis identified statistically significant pathways with a p-value <0.05. Enrichment score reflects the degree to which a pathway is overrepresented at the top or bottom of the ranked list of proteins. Gene Enrichment analysis was completed using the Molecular Signature Database all proteins. 78 pathways were significantly enriched for AC6 differentially regulated proteins.

|  | Enrichmentscore | Normalized Enrichment score | P value |
| --- | --- | --- | --- |
| GOBP_CELL_CYCLE_PROCESS | -0.8417732 | -2.3123539 | 0.00172058 |
| GOBP_POSITIVE_REGULATION_OF_CELLULAR_COMPONENT_BIOGENESIS | -0.9354839 | -1.9349546 | 0.00468994 |
| GOBP_CELLULAR_MACROMOLECULE_LOCALIZATION | -0.7931034 | -2.0367932 | 0.00507292 |
| GOBP_MITOTIC_CELL_CYCLE_PROCESS | -0.7818259 | -2.007831 | 0.00507292 |
| GOBP_POSITIVE_REGULATION_OF_IMMUNE_SYSTEM_PROCESS | 0.96875 | 1.58295611 | 0.00539224 |
| GOBP_TAXIS | 0.96875 | 1.58295611 | 0.00539224 |
| GOMF_TRANSITION_METAL_ION_BINDING | 0.96875 | 1.58295611 | 0.00539224 |
| KEGG_PATHWAYS_IN_CANCER | 0.96875 | 1.58295611 | 0.00539224 |
| GOBP_AMIDE_BIOSYNTHETIC_PROCESS | -0.9032258 | -1.868232 | 0.00807712 |
| GOBP_CELLULAR_AMIDE_METABOLIC_PROCESS | -0.9032258 | -1.868232 | 0.00807712 |
| GOBP_ORGANONITROGEN_COMPOUND_BIOSYNTHETIC_PROCESS | -0.9032258 | -1.868232 | 0.00807712 |
| GOBP_PEPTIDE_BIOSYNTHETIC_PROCESS | -0.9032258 | -1.868232 | 0.00807712 |
| GOBP_PEPTIDE_METABOLIC_PROCESS | -0.9032258 | -1.868232 | 0.00807712 |
| GOBP_REGULATION_OF_TRANSPORT | -0.8333333 | -1.9399969 | 0.0094284 |
| GOMF_MOLECULAR_ADAPTOR_ACTIVITY | -0.8333333 | -1.9399969 | 0.0094284 |
| GSE29618_LAIV_VS_TIV_FLU_VACCINE_DAY7_PDC_UP | -0.96875 | -1.7181967 | 0.01304646 |
| MIR3065_3P | -0.96875 | -1.7181967 | 0.01304646 |
| CBX5_TARGET_GENES | -0.9375 | -1.662771 | 0.01739528 |
| DLX4_TARGET_GENES | -0.9375 | -1.662771 | 0.01739528 |
| FAN_OVARY_CL14_MATURE_SMOOTH_MUSCLE_CELL | -0.9375 | -1.662771 | 0.01739528 |
| GOBP_REGULATION_OF_ACTIN_FILAMENT_BASED_PROCESS | -0.9375 | -1.662771 | 0.01739528 |
| GOBP_REGULATION_OF_ACTIN_FILAMENT_ORGANIZATION | -0.9375 | -1.662771 | 0.01739528 |
| GOBP_REGULATION_OF_ANATOMICAL_STRUCTURE_SIZE | -0.9375 | -1.662771 | 0.01739528 |
| GOCC_MEMBRANE_PROTEIN_COMPLEX | -0.9375 | -1.662771 | 0.01739528 |
| GOCC_RUFFLE | -0.9375 | -1.662771 | 0.01739528 |
| GOTZMANN_EPITHELIAL_TO_MESENCHYMAL_TRANSITION_DN | -0.9375 | -1.662771 | 0.01739528 |
| GSE29164_UNTREATED_VS_CD8_TCELL_AND_IL12_TREATED_MELANOMA_DAY3_DN | -0.9375 | -1.662771 | 0.01739528 |
| GSE29618_PRE_VS_DAY7_POST_TIV_FLU_VACCINE_MDC_DN | -0.9375 | -1.662771 | 0.01739528 |
| GSE33425_CD161_INT_VS_NEG_CD8_TCELL_DN | -0.9375 | -1.662771 | 0.01739528 |
| LAKE_ADULT_KIDNEY_C28_INTERSTITIUM | -0.9375 | -1.662771 | 0.01739528 |
| RUBENSTEIN_SKELETAL_MUSCLE_SMOOTH_MUSCLE_CELLS | -0.9375 | -1.662771 | 0.01739528 |
| MIR4699_3P | 0.87096774 | 1.59394787 | 0.01767068 |
| RBM34_TARGET_GENES | -0.7666667 | -1.7847972 | 0.01915144 |
| GOBP_RESPONSE_TO_OXYGEN_CONTAINING_COMPOUND | -0.6296296 | -1.8376713 | 0.01976137 |
| GOCC_CILIUM | 0.85803691 | 1.57028331 | 0.01991968 |
| GSE1925_CTRL_VS_IFNG_PRIMED_MACROPHAGE_24H_IFNG_STIM_UP | -0.7666667 | -1.7847972 | 0.02268709 |
| BLALOCK_ALZHEIMERS_DISEASE_INCIPIENT_UP | 0.83819495 | 1.53397077 | 0.02955823 |
| GOBP_NEGATIVE_REGULATION_OF_NUCLEOBASE_CONTAINING_COMPOUND_METABOLIC_PROCESS | 0.90625 | 1.48082991 | 0.03096191 |
| GOBP_REGULATION_OF_CELLULAR_RESPONSE_TO_STRESS | 0.90625 | 1.48082991 | 0.03096191 |
| GOBP_REGULATION_OF_RESPONSE_TO_DNA_DAMAGE_STIMULUS | 0.90625 | 1.48082991 | 0.03096191 |
| GOBP_REGULATION_OF_RESPONSE_TO_STRESS | 0.90625 | 1.48082991 | 0.03096191 |
| GOCC_NUCLEOLUS | 0.90625 | 1.48082991 | 0.03096191 |
| MIR3182 | 0.90625 | 1.48082991 | 0.03096191 |
| MIR3619_5P | 0.90625 | 1.48082991 | 0.03096191 |
| MIR506_5P | 0.90625 | 1.48082991 | 0.03096191 |
| GOBP_REGULATION_OF_IMMUNE_SYSTEM_PROCESS | 0.78469098 | 1.55787376 | 0.03368421 |
| GOCC_MICROTUBULE_ORGANIZING_CENTER | -0.7333333 | -1.7071973 | 0.03476724 |
| FEVR_CTNNB1_TARGETS_UP | -0.90625 | -1.6073453 | 0.03730831 |
| GOBP_CELL_JUNCTION_ASSEMBLY | -0.90625 | -1.6073453 | 0.03730831 |
| GOBP_CELL_JUNCTION_ORGANIZATION | -0.90625 | -1.6073453 | 0.03730831 |
| GOBP_ENDOCYTOSIS | -0.90625 | -1.6073453 | 0.03730831 |
| GOBP_POSITIVE_REGULATION_OF_CELL_JUNCTION_ASSEMBLY | -0.90625 | -1.6073453 | 0.03730831 |
| GOBP_POSITIVE_REGULATION_OF_CELL_PROJECTION_ORGANIZATION | -0.90625 | -1.6073453 | 0.03730831 |
| GOBP_POSITIVE_REGULATION_OF_TRANSPORT | -0.90625 | -1.6073453 | 0.03730831 |
| GOBP_REGULATION_OF_CELL_JUNCTION_ASSEMBLY | -0.90625 | -1.6073453 | 0.03730831 |
| GOBP_REGULATION_OF_ENDOCYTOSIS | -0.90625 | -1.6073453 | 0.03730831 |
| GOBP_REGULATION_OF_VESICLE_MEDIATED_TRANSPORT | -0.90625 | -1.6073453 | 0.03730831 |
| GOBP_VESICLE_MEDIATED_TRANSPORT | -0.90625 | -1.6073453 | 0.03730831 |
| GSE1925_CTRL_VS_24H_IFNG_STIM_IFNG_PRIMED_MACROPHAGE_UP | -0.90625 | -1.6073453 | 0.03730831 |
| ZWANG_CLASS_1_TRANSIENTLY_INDUCED_BY_EGF | -0.90625 | -1.6073453 | 0.03730831 |
| GOBP_CELLULAR_RESPONSE_TO_ENDOGENOUS_STIMULUS | -0.8064516 | -1.6680643 | 0.03856175 |
| GOBP_CELLULAR_RESPONSE_TO_HORMONE_STIMULUS | -0.8064516 | -1.6680643 | 0.03856175 |
| GOBP_CELLULAR_RESPONSE_TO_NITROGEN_COMPOUND | -0.8064516 | -1.6680643 | 0.03856175 |
| GOBP_CELLULAR_RESPONSE_TO_PEPTIDE | -0.8064516 | -1.6680643 | 0.03856175 |
| GOBP_CELLULAR_RESPONSE_TO_PEPTIDE_HORMONE_STIMULUS | -0.8064516 | -1.6680643 | 0.03856175 |
| GOBP_RESPONSE_TO_ENDOGENOUS_STIMULUS | -0.8064516 | -1.6680643 | 0.03856175 |
| GSE18281_SUBCAPSULAR_CORTICAL_REGION_VS_WHOLE_CORTEX_THYMUS_UP | 0.875 | 1.4297668 | 0.04574709 |
| GSE37301_RAG2_KO_VS_RAG2_AND_ETS1_KO_NK_CELL_UP | 0.875 | 1.4297668 | 0.04574709 |
| GSE4984_GALECTIN1_VS_VEHICLE_CTRL_TREATED_DC_UP | 0.875 | 1.4297668 | 0.04574709 |
| MIR4510 | 0.875 | 1.4297668 | 0.04574709 |
| MIR492 | 0.875 | 1.4297668 | 0.04574709 |
| MIR544B | 0.875 | 1.4297668 | 0.04574709 |
| MIR6127 | 0.875 | 1.4297668 | 0.04574709 |
| MIR6129 | 0.875 | 1.4297668 | 0.04574709 |
| MIR6130 | 0.875 | 1.4297668 | 0.04574709 |
| MIR6133 | 0.875 | 1.4297668 | 0.04574709 |
| MEISSNER_BRAIN_HCP_WITH_H3K4ME3_AND_H3K27ME3 | 0.76389615 | 1.51658908 | 0.04947368 |
| GSE13547_WT_VS_ZFX_KO_BCELL_UP | 0.8629054 | 1.410004 | 0.04957384 |

**Supplemental Table 3: List of kinases and the number of significantly up and down regulated phosphopeptides in AC2 and AC6 samples.** We analyzed each significantly altered phosphorylated sequence (a fold change of 1.5 or 1/1.5, P<0.05) using Scansite 4.0 to identify the kinases capable of phosphorylating that sequence. The table shows the number of significant phosphopeptides that could be regulated by each kinase in each condition.

| **Kinases** | **AC2 Up** | **AC2 Down** | **AC6 Up** | **AC6 Down** |
| --- | --- | --- | --- | --- |
| 14-3-3 mode 1 | 3 | 1 | 8 | 1 |
| Abl_sh3 | - | 1 | - | - |
| AKT | 2 | - | 9 | 1 |
| ATK | 1 | - | 1 | - |
| AMP | 2 | 1 | 4 | 1 |
| ATM | - | - | 1 | - |
| Aurora A | 3 | 1 | 12 | 2 |
| Aurora B | 4 | 2 | 11 | 2 |
| Cam_Kin (CAMK1) | - | - | 2 | 1 |
| Cam_Kin2 (CAMK2) | - | - | 2 | - |
| Casn_kin1 (CK1) | 1 | 1 | 1 | - |
| Casn_kin2 (CK2) | 1 | 1 | 2 | - |
| CDC2 | - | 3 | 3 | 1 |
| CDK1 Mode 1 (CDK1_1) | - | 3 | 2 | 2 |
| CDK1 Mode 2 (CDK1_2) | - | 3 | 2 | 1 |
| CDK5 | - | 3 | 3 | 1 |
| CLK2 | - | 1 | 6 | 1 |
| DNA_PK | - | 1 | 2 | - |
| ERK1 | 1 | 1 | 4 | 1 |
| ERKDD | - | 1 | - | - |
| GSK3 | 1 | 1 | 6 | - |
| NCK-2nd_sn3 | - | 1 | - | - |
| NEK10 | - | - | 1 | - |
| NEK1 | - | - | 1 | - |
| NEK2 | - | - | 1 | - |
| NEK3 | - | - | 2 | - |
| NEK4 | - | - | 2 | - |
| NEK5 | - | 1 | - | - |
| NEK6 | 1 | 1 | 2 | 1 |
| Nek7 | - | 2 | 1 | 1 |
| NEK8 | - | 1 | - | - |
| NEK9 | - | - | 1 | - |
| PDK 1 Binding | - | - | 2 | - |
| PKA | 4 | 2 | 8 | 1 |
| PKA α,β,γ | 1 | - | - | 2 |
| PKC | - | - | 1 | - |
| PKC common | - | - | 1 | - |
| PKC δ | 1 | - | 1 | 1 |
| PKC ε |  | 1 | 3 | 1 |
| PKC μ | 1 | - | 2 | - |
| PLK ε | - | 1 | - | - |
| PLK1 | - | 2 | 1 | - |
| P38 MAPK | - | - | - | 1 |
| **TOTAL** | **27** | **37** | **112** | **23** |
